# Hsa_circ_0046263 functions as a ceRNA to promote nasopharyngeal carcinoma progression by upregulating IGFBP3

**DOI:** 10.1038/s41419-020-02785-3

**Published:** 2020-07-23

**Authors:** Li Yin, Jie Chen, Chengxian Ma, Shuai Pei, Mingyu Du, Yufeng Zhang, Yong Feng, Rong Yin, Xiuhua Bian, Xia He, Jifeng Feng

**Affiliations:** 1 The Affiliated Cancer Hospital of Nanjing Medical University, Jiangsu Cancer Hospital, Jiangsu Institute of Cancer Research, Nanjing, Jiangsu China; 2 Xuzhou Medical University, Xuzhou, Jiangsu China

**Keywords:** Oncogenes, Cell migration, Diagnostic markers

## Abstract

Accumulating evidences indicate that circular RNAs (circRNAs), a subclass of noncoding RNAs, play important role in regulating gene expression in eukaryotes. Hsa_circ_0046263 (circ-0046263) was found aberrantly expressed in nasopharyngeal carcinoma (NPC), but its role in tumor growth and metastasis remains largely unclear. Sanger sequencing, RNase R assay, and nucleic acid electrophoresis were conducted to verify the identification of circ-0046263. Nuclear separation and fluorescence in situ hybridization (FISH) assays were used to determine the localization of circ-004263. Dual luciferase reporter and RNA immunoprecipitation (RIP) were employed to confirm the binding of circ-0046263 with miR-133a-5p. Colony formation, proliferation, wound healing, transwell, western blot, and in vivo tumor growth and metastasis assays were performed to assess the roles of circ-0046263, miR-133a-5p, IGFBP3 and their interactions in NPC cells. Circ-0046263 was upregulated in both NPC cell lines and tissues. The in vitro functional studies revealed that knockdown of circ-0046263 inhibited the proliferation, invasion, and migration of NPC cells, whereas its overexpression produced the opposite result. In vivo experiments indicated that knockdown or overexpression of circ-0046263 attenuated or promoted tumor growth and metastasis, respectively. Mechanistically, circ-0046263 could act as a miRNA sponge to absorb miR-133a-5p and upregulate the expression of miRNA downstream target IGFBP3. In addition, miR-133a-5p inhibition or IGFBP3 overexpression could rescue the malignant behavior induced by circ-0046263 silencing. Finally, circ-0046263 plays a tumor-promoting role in NPC to enhance malignant behavior through the miR-133a-5p/IGFBP3 axis, which could be a potential target for NPC therapy.

## Introduction

Nasopharyngeal carcinoma (NPC) is a malignant tumor that originates from the superior mucosal epithelium of the nasopharynx. It is characterized by unique geographic distribution and is primarily prevalent in east and southeast Asian, with the highest incidence in southeast China^1,2^. At present, radiation is the most common therapy for NPC. With the advances in radiotherapy technology, especially high-precision image-guided radiation therapy, the treatment of NPC has achieved favorable outcomes^1,3^.

However, local recurrence and distant metastasis are very common in advanced-stage patients with NPC^4^. It is estimated that more than 30% of patients of stages III and IV will develop local recurrence or distant metastasis within 5 years after receiving combined treatment^4,5^. Thus, further understanding of the molecular mechanisms underlying NPC progression and metastasis and identifying potential therapeutic targets are of paramount importance.

Circular RNAs are a class of new noncoding RNA characterized by covalently closed loop structure that lack 5ʹ and 3ʹ polarities and polyadenylate tails^6^. These molecules are stable and widely expressed in tissues during different developmental stages in various organisms. Numerous studies have demonstrated that circRNAs mainly function by sponging microRNA, binding to RNA-binding proteins, or translating proteins^7^. Growing evidence has indicated that circRNAs play important roles in tumors, and their dysregulation may contribute to tumorigenesis and progression^8^. It has been reported that circRNAs are involved in multiple processes, including proliferation, epithelial–mesenchymal transition (EMT), apoptosis, and cell cycle regulation^9,10^. However, the roles and mechanism of most circRNAs in NPC are not fully characterized.

In this study, we identified a circRNA, namely, circ-0046263, which is expressed in NPC tissues and appears to regulate NPC migration and invasion. We found that the expression of circ-0046263 was upregulated in both cell lines and NPC tissues. We aimed to investigate the regulatory mechanisms of circ-0046263 in NPC growth and metastasis. Results showed that circ-0046263 was a negative NPC metastatic factor. Importantly, we reported that miR-133a-5p targeted IGFBP3 directly and circ-0046263 acted as a sponge for miR-133a-5p and thus upregulated the expression of IGFBP3. Thus, circ-0046263 promoted NPC growth and metastasis by regulating the miR-133a-5p/IGFBP3 axis. Our findings revealed that circ-0046263 could be a prognostic factor and a promising target for NPC treatment.

## Materials and methods

### Clinical specimens

Forty frozen NPC tissues (stages I and II: 11 patients; stage III: 13 patients; stage IV: 16 patients) and 8 normal nasopharyngeal epithelium tissues were obtained from patients treated in Jiangsu Cancer Hospital (Nanjing, China). All tissue samples were confirmed by pathologists. This study was approved by the Institutional Ethical Review Committee of Jiangsu Cancer Hospital, and each patient provided written informed consent.

### Cell culture

Jiangsu Cancer Hospital provided seven human NPC cell lines (6-10B, CNE-1, CNE-2, SUNE-1, 5–8F, HNE-1, and C666-1) and a human immortalized nasopharyngeal epithelial cell line (NP69). The seven human NPC cells were incubated in RPMI-1640 medium (Corning) containing 10% FBS. NP69 was cultured in keratinocyte/serum-free medium covered in growth factors (Gibco, Grand Island, NY, USA). All cell lines were cultured in humidified air at 37 °C with 5% CO_2._ All the cell lines were approved by the Institutional Ethical Review Board of Jiangsu Cancer Hospital. In addition, the cell lines we used were authenticated by STR profile. All cells were tested negative for mycoplasma contamination.

### RNA extraction, gDNA extraction, and quantitative real-time PCR (qRT-PCR)

Total RNA from clinical samples or NPC cells was extracted using TRIzol reagent (Invitrogen). The mammalian genomic DNA extraction kit (Beyotime D0061) was used to extract genomic DNA (gDNA) from the cultured cells. For the RNase R treatment, 5 μg of total RNA was incubated for 30 min at 37 °C with or without 3 U/μg RNase R (GENESEED R0301). SYBR Green PCR Master Mix was used for qRT-PCR reactions on a 7500 FAST real-time PCR machine (Applied Biosystems). β-actin, U6, and GAPDH were used as standardized controls. The sequences of all primers are listed in Table S1.

### Nucleic acid electrophoresis

gDNA and cDNA PCR products were examined using 2% agarose gel electrophoresis and TAE running buffer. The DNA was separated by 160 V electrophoresis for 15 min. The DNA marker used was DL2000 (KeyGen, Nanjing), and the strip was checked by UV irradiation.

### Subcellular fractionation location

The PARIS kit (Invitrogen) was used according to the manufacturer’s guidelines to detect the subcellular localization of circ-0046263.

### Fluorescence in situ hybridization (FISH)

5–8F and CNE-2 cells were fixed in 4% formaldehyde for 30 min and then permeabilized with Triton X-100. The cells were incubated with a hybridization buffer containing the FISH probe (RiboBio) and washed with 2× saline-sodium citrate. FISH Kit (RiboBio, Guangzhou, China) was used to detect signals according to the manufacturer’s protocol. Images were taken using an Olympus confocal laser scanning microscope.

### RNA interference and stable transfection

SiRNAs, miRNA mimics, inhibitors, and primers were provided by RiboBio. SUNE-1 cells were infected with lentivirus containing the circ-0046263 plasmid synthesized by GeneChem (Shanghai, China). Transfection experiments were performed according to the instructions of Lipofectamine 3000 (Invitrogen). All sequences are listed in Table S1.

### Colony formation and cell viability assays

The transfected cells were seeded into a six-well plate (1000 cells/well) and then cultured for 14 days. After the cells were fixed with 2% paraformaldehyde and stained with 0.5% crystal violet for 1 h, the number of colonies was counted by using Image J. Cell viability was measured by the CCK-8 assay (Promega). The transfected cells were seeded in a 96-well plate (3000 cells/well). CCK-8 reagent was added into the cultured cells, and each well was detected with a spectrophotometer at 450 nm after being cultured for 1 h.

### Wound-healing assay

Wound-healing assay was performed to determine the migration ability. After transfection, the cells were placed in a six-well plate and cultured for 48 h with serum-free medium. A wound was created artificially by using a 200 μL pipette tube. Cell migration was measured at ×100 magnification under light microscopy at 0 and 24 h.

### Transwell migration and invasion assay

Transwell chambers with or without Matrigel cover (BD Biosciences) were used to evaluate cell migration and invasion potential. The transfected cells were resuspended (3 × 10^4^ cells per well) into 200 μL of serum-free medium and plated in the upper chamber. Then, 20% FBS and 500 μL of RPMI-1640 were added to the lower compartment. After 36 h of incubation, the upper chamber was fixed and stained. An inverted microscope was used to count the invading cells.

### Luciferase reporter assay

A circ-0046263 segment was synthesized with either a mutant (Mut) or a wild-type (Wt) seed region and cloned into the pcDNA3.1 luciferase reporter vector. The NPC cells were cotransfected with Wt or mutated (Mut) circ-0046263 and miRNA mimic or mimic control using Lipofectamine 3000 (Invitrogen). After induction for 48 h, luciferase activity was assessed using the dual-luciferase reporter kit (Promega, Madison, USA). The IGFBP3 Wt and Mut 3′-UTR were constructed and cloned into a pcDNA3.1 luciferase reporter vector. The NPC cells were cotransfected with IGFBP3 Wt or Mut 3′-UTR vector and miR-133a-5p mimic or mimic NC by using Lipofectamine 3000 reagent (Invitrogen). In total, 2 μg of a specific plasmid and mimic were co-transfected in NPC cells. After 48 h, renilla and firefly luciferase activities was assessed using the Dual Luciferase Assay Kit (Promega) according to the Kit instructions.

### RNA immunoprecipitation (RIP)

RIP assay was performed with Magna RIP Kit (Billerica, MA, USA). The NPC cells were lysed in complete RIP lysis buffer, and the cell extracts were incubated with magnetic beads conjugated with anti-argonaute 2 (AGO2) or control anti-IgG antibody for 6 h at 4 °C. Proteinase K was incubated to remove proteins after the magnetic beads were washed. The purified RNA was used for qRT-PCR analysis.

### Western blot analysis

Proteins were extracted from the transfected cell lines by using modified RIPA buffer and PMSF (Beyotime, Shanghai) following the manufacturer’s protocols. BCA protein assay kit (Beyotime, Shanghai) was used to quantify the protein concentration. Protein (10 mg) from each sample was separated using 10% SDS-PAGE gels and then transferred onto a nitrocellulose membrane. The membranes were incubated with specific antibodies. Primary antibodies used were listed as follows: P4HB (Abcam, ab137110), IGFBP3 (Cell Signaling Technology, 25864, USA), E-cadherin (Cell Signaling Technology, 3195, USA), N-cadherin (Cell Signaling Technology, 13116, USA), Vimentin (Cell Signaling Technology, 5741, USA), GAPDH (Cell Signaling Technology, 5174, USA), β-actin (Cell Signaling Technology, 4970, USA), Smad2 (Cell Signaling Technology, 5339, USA), Smad3 (Cell Signaling Technology, 9523, USA). The immunoreactive bands were imaged with ECL detection reagents (Millipore, Billerica, MA, USA).

### In vivo tumor growth and metastasis assays

Six-week-old immunodeficient male BALB/c nude mice were obtained from GemPharmatech Co, Ltd (Jiangsu, China), and were randomly divided into four groups, with 6 mice in each group. 5–8F cells expressing green fluorescent protein (GFP) were injected into the footpad of the nude mice in the G1 and G2 groups. When the volume of the tumor reached 100 mm3, si-circ-0046263 or si-NC was injected into plantar xenograft tumors. In the G3 and G4 groups, 20 μL of cell suspension of 2 × 10^6^ SUNE-1 cells infected with lentivirus containing circ-0046263 expression vector or empty vector were injected into the footpad of the nude mice. The volume of the tumor was measured every 3–4 days and calculated using the following formula: *V* = *ab*^2^/2, where *V* is the volume, *a* is the longitudinal diameter, and *b* is the lateral diameter. After 4–6 weeks of injection, all mice were sacrificed and dissected. The xenograft tumors were weighed. Mice with axillary lymph node metastasis were counted and confirmed with GFP fluorescence. Animal experiments were conducted in accordance with the Institute for Laboratory Animal Research Guide for the Care and Use of Laboratory Animals and followed protocols approved by the Animal Science Committee of Origin, Nanjing, China.

### Immunohistochemistry

Formalin-fixed and paraffin-embedded tissue sections were incubated with primary antibody at 4 °C overnight, and then the kit (ZSGB BIO Inc.) was used for the DAB chromogen followed by nuclear staining using haematoxylin.

### Statistical analysis

Statistical analysis was performed using Prism 7 (San Diego, CA, USA) and SPSS 23.0 (Chicago, IL, USA) software, including ANOVA and Student’s *t* test. The experiments were independently repeated three times. Data were expressed as mean ± SD. *P* values < 0.05 were considered significant (*P* < 0.05, *P* < 0.01 and *P* < 0.001).

## Results

### Circ-0046263 was relatively highly expressed in NPC tissues and cell lines

**Fig. 1:**
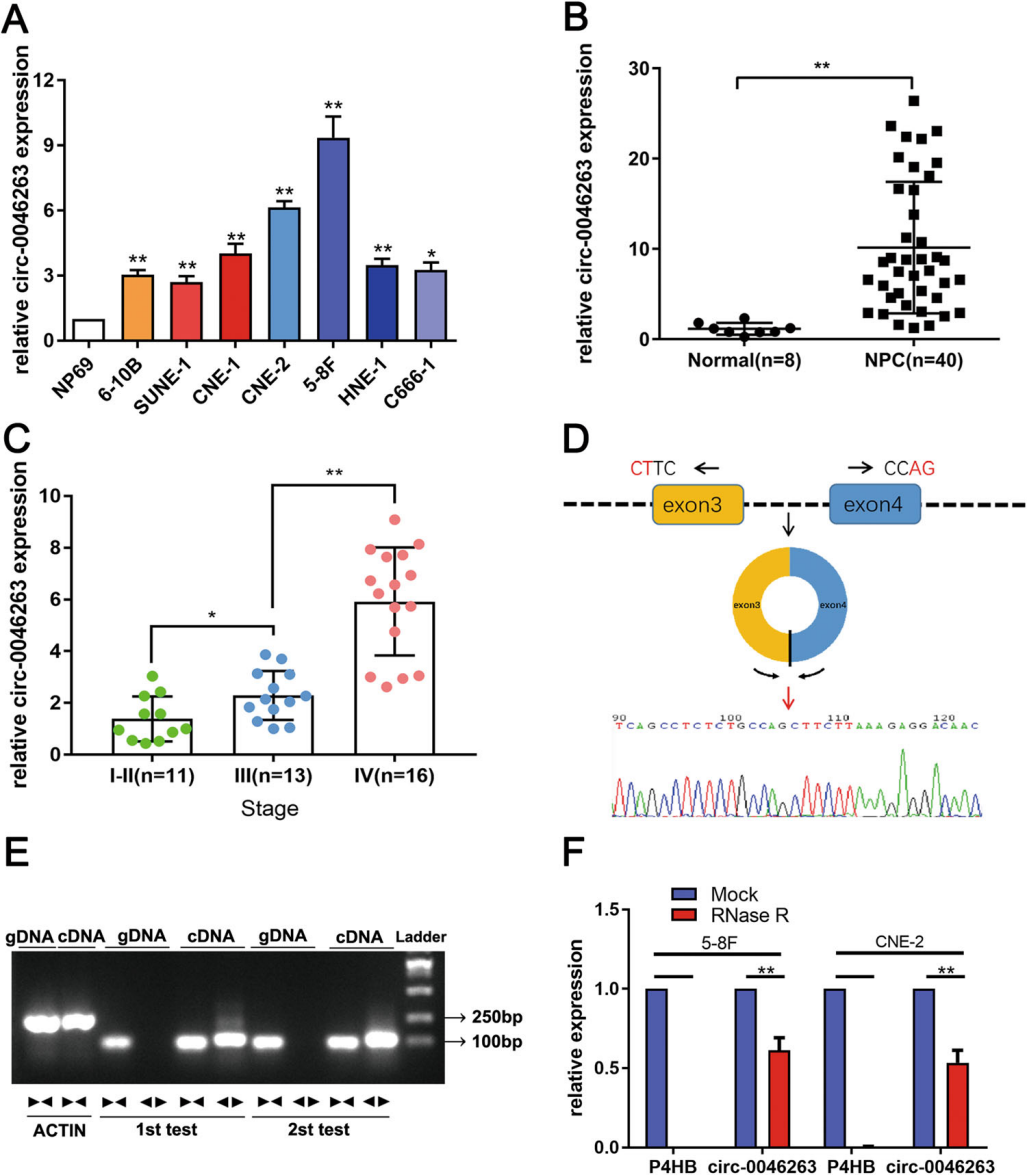
Expression and validation of circ-0046263 in NPC cells and tissues. **a** Circ-0046263 expression in NPC cell lines and nasopharyngeal epithelial cell line NP69. **b** Relative circ-0046263 expression in normal nasopharyngeal epithelial tissues (*n* = 8) and NPC tissues (*n* = 40). **c** Relative expression of circ-046263 in different clinical stages. **d** Circ-0046263 was formed by the cyclization of exons 3 and 4. The red arrow indicates the head-to-tail stitch position of circ-0046263. **e** Divergent primers amplified circ-0046263 in cDNA but not in gDNA. **f** qRT-PCR was performed to analyze the circ-0046263 and P4HB level after treatment with RNase R. Circ-0046263 was resistant to RNase R treatment. Results are shown as mean ± SD, and the data were represented by three independent experiments. *P < 0.05; **P < 0.01.

To investigate circRNA expression in NPC tissues, we analyzed five pair of NPC tissue samples by using circRNA expression microarray and found a cluster of differentiated expressed circRNAs. Among them, we found that the expression level of circ-0046263 was consistently and significantly increased in NPC tumor tissues compared to the matched nontumor tissues. qRT-PCR shown that circ-0046263 expression was increased in various NPC cell lines compared with that in immortalized nasopharyngeal epithelial NP69 cells (Fig. 1a). As shown in Fig. 1b, compared with corresponding normal tissues, tumor tissues (*n* = 40) displayed significantly increased level of circ-0046263. Furthermore, we investigated the relationship between circ-0046263 expression level and the clinic feature pathological characteristics in 40 NPC patients. The expression of circ-0046263 was positively correlated with the clinical pathological stage of NPC (Fig. 1c). Further statistical analysis showed that the level of circ-0046263 was significantly associated with lymph node metastasis and distant metastasis but not with age or gender (Table 1). Validation results revealed that circ-0046263 was circular and contained exons 3 and 4 of its host gene P4HB by comparing the P4HB mRNA sequence with the expected sequence of circ-0046263 obtained from circBase. To further characterize circ-0046263, we performed Sanger sequencing to confirm head-to-tail splicing (Fig. 1d). However, head-to-tail splicing may be the result of transsplicing or genomic rearrangement. Therefore, we designed a convergent primer for P4HB mRNA and a special divergent primer for circ-0046263 to distinguish these two possibilities. cDNA and gDNA were extracted from 5 to 8F cells. Nucleic acid electrophoresis results showed that circ-0046263 was amplified only in the cDNA using the divergent primer, as no products were amplified in the gDNA (Fig. 1e). The stability of circ-0046263 was confirmed using RNase R. The results indicated that the linear transcript of P4HB was degraded by RNase R, whereas the circular transcript of circ-0046263 was resistant to RNase R treatment (Fig. 1f). In summary, these results confirmed the presence of circ-0046263 and its possible tumor-promoting function in NPC.

**Table 1:** Association of circ-0046263 level with clinicopathologic features in NPC.

Characteristics	Case	Relative circ-0046263 expression average	*P* value
Age			
<55	23	3.4133	0.8631
≥55	17	3.4133	
Gender			
Male	27	3.7973	0.2703
Female	13	2.8696	
TNM stage			
I + II	11	1.3850	<0.001*
III + IV	29	4.2965	
Node metastasis			
N0	6	1.8045	0.0028*
N1–N2	34	3.7943	
Distant metastasis			
M0	35	3.1441	0.0264*
M1	5	5.9580	

*P < 0.05, statistically significant

### NPC cell proliferation, invasion, and migration regulation by circ-0046263 in vitro

**Fig. 2:**
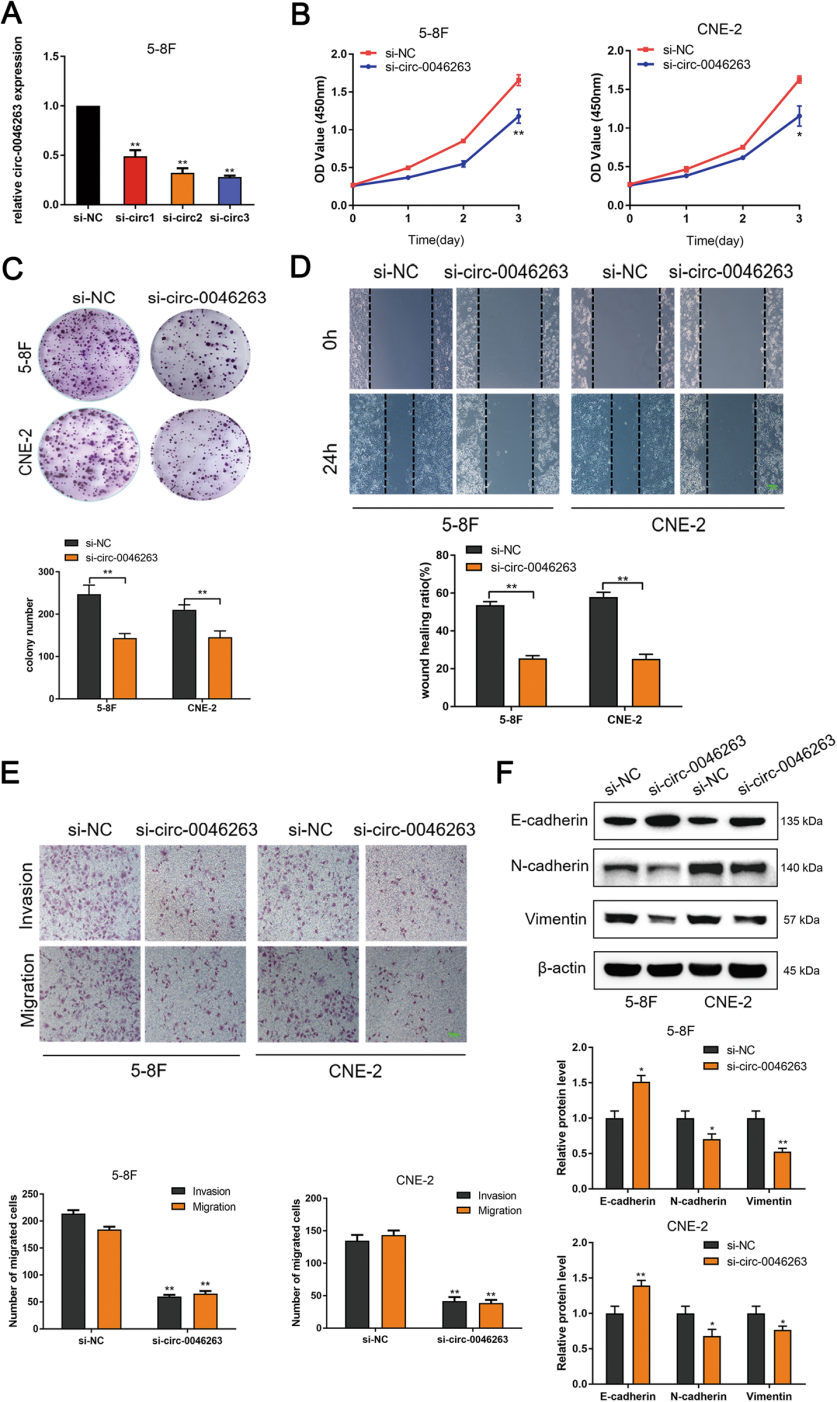
NPC cell proliferation, invasion, and migration regulation by circ-0046263 in vitro. **a** qRT-PCR analysis of circ-0046263 expression after treatment with three siRNAs. **b** CCK-8 assay was performed to assess NPC cell growth vitality. **c** The proliferative capacity in 5–8F and CNE-2 cells were determined via colony-formation assay. **d**, **e** Wound-healing assay and Transwell assay were performed to assess migration and invasion ability of cells transfected with si-circ-0046263 or si-NC. Scale bars, 100 μm. **f** Expression of EMT-related proteins was detected by Western blot analysis. Results are shown as mean ± SD, and the data were represented by three independent experiments. *P < 0.05; **P < 0.01.

In order to explore the biological function of circ-0046263 in vitro, we selected three representative cells (5–8F, CNE-2, and SUNE-1) for research. Circ-0046263 small interfering RNA (siRNA) could knockdown the circ-0046263 expression in NPC cells. We designed three siRNA products and selected the siRNA3 with the best knockdown efficiency for further study (Fig. 2a). Compared with the control group, circ-0046263 knockdown significantly inhibited the proliferation, invasion, and migration of NPC cells in vitro (Fig. 2b–e). However, circ-0046263 overexpression significantly increased the proliferation, invasion, and migration abilities of NPC cells (Fig. S1A–E). Western blot analysis was performed to analyze the EMT-related protein levels. The results showed that the expression level of E-cadherin was significantly increased in the downregulated circ-0046263 cells, whereas the vimentin and N-cadherin levels were significantly decreased (Fig. 2f). The opposite effect was produced in the cells stably overexpressing circ-0046263 (Fig. S1F). In addition, after overexpressing circ-0046263 in NPC cells, we observed through phase contrast microscopy that the cells became elongated and scattered (Fig. S1G).

### Circ-0046263 functioned as an efficient miR-133a-5p sponge

**Fig. 3:**
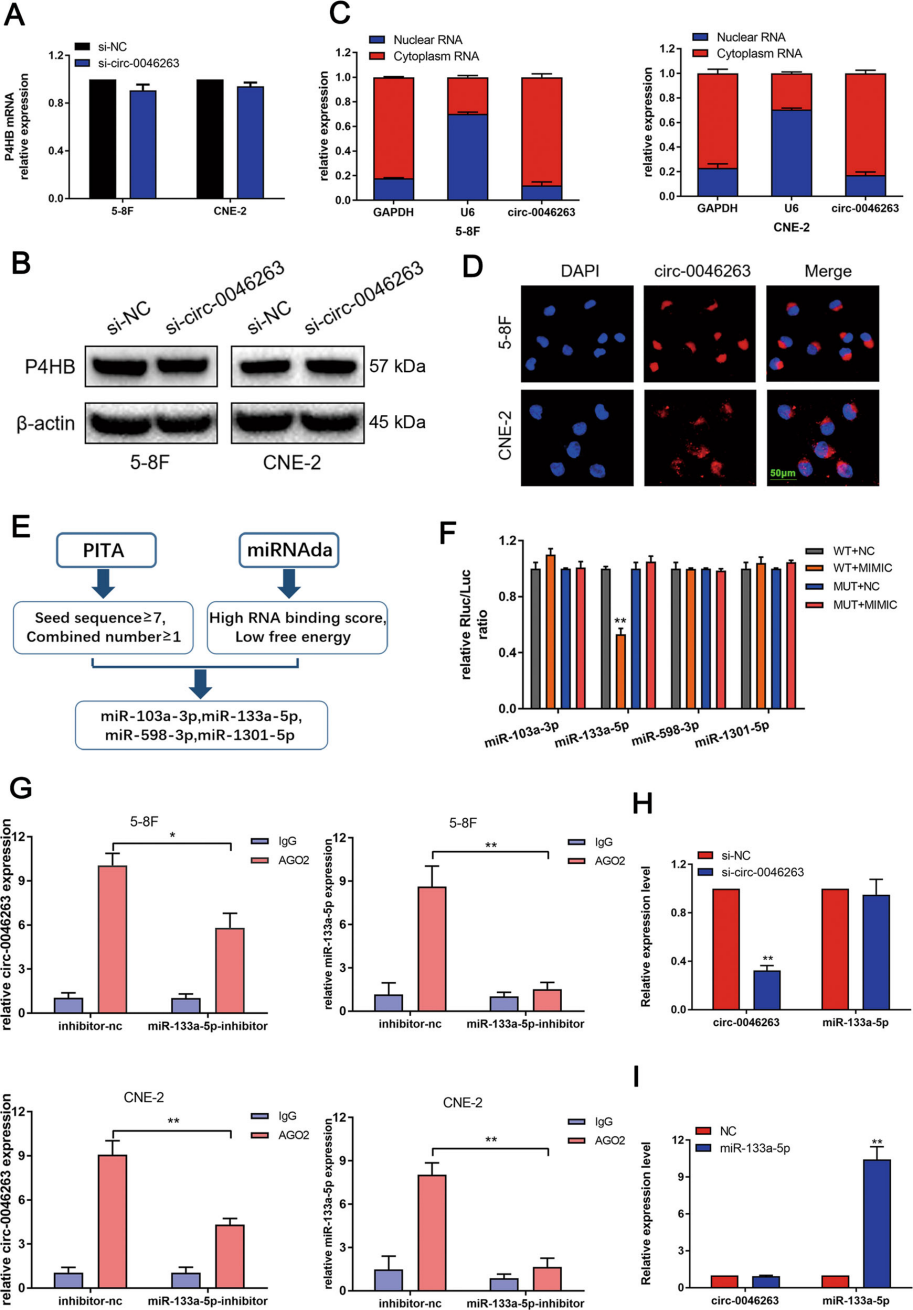
Circ-0046263 functions as a miRNA sponge in NPC cells. **a**, **b** mRNA and protein expressions of the host gene were detected by qRT-PCR and Western blot after knocking down circ-00466263. **c**, **d** Nuclear separation and FISH shown subcellular localization of circ-0046263. Scale bars, 50 μm. **e** PITA and miRnada predicted miRNAs targeting circ-0046263. **f** Luciferase reporter assay detected luciferase activity after co-transfecting with four miRNA mimics and circ-0046263-WT or circ-0046263-MUT reporter plasmid. **g** miR-133a-5p was identified in the circ-0046263-RISC complex. Control and miR-133a-5p-inhibitor cell lysates were used for RNA-IP with anti-AGO2 antibody. Circ-0046263 and miR-133a-5p expression levels were detected by qRT-PCR. **h**, **i** Silencing of circ-0046263 did not affect the expression of miR-133a-5p, and miR-133a-5p did not affect the expression of circ-0046263. All data are presented as mean ± SD of at least three independent experiments. *P < 0.05; **P < 0.01.

We examined the relationship between circ-0046263 and its host gene P4HB and found that circ-0046263 knockdown had no significant effect on P4HB mRNA and protein levels (Fig. 3a, b). Nuclear separation assay (Fig. 3c) and FISH analysis (Fig. 3d) were conducted to determine the subcellular localization of circ-0046263 in NPC cell lines. We found that circ-0046263 was mainly localized in the cytoplasm. Previous studies reported that many circRNAs in the cytoplasm can act as miRNA sponges^11^. We determined whether circ-0046263 can also act as miRNA sponges and regulate targets. Therefore, we used miRanda and PITA algorithms to analyze the sequence of circ-0046263. Four miRNAs with relatively high scores were identified (miR-103a-3p, miR-133a-5p, miR-598-3p, and miR-1301-5p) (Fig. 3e). Wt and Mut circ-0046263 were constructed for the dual luciferase reporter assay. Only miR-133a-5p mimics effectively inhibited the luciferase activity of the Wt group compared with the other groups. However, luciferase activity was restored after the binding sites were mutated (Fig. 3f). miRNAs typically form RNA-induced silencing complex (RISC) via binding to the AGO2 protein, and this phenomenon may be a common occurrence^12,13^. Therefore, RIP assay was conducted to pull down RNA transcripts that bind to AGO2 in 5–8F and CNE-2 cells. As shown in Fig. 3g, AGO2 effectively pulled down circ-0046263 and miR-133a-5p compared with the corresponding IgG group. The levels of circ-0046263 and miR-133a-5p immunoprecipitated with AGO2 were lower in the NPC cells transfected with miR-133a-5p-inhibitor than those in the control group. However, a high level of circ-0046263 was immunoprecipitated by AGO2 compared with the IgG control. By contrast, the amount of miR-133a-5p immunoprecipitated with AGO2 was statistically indistinguishable from that immunoprecipitated with IgG (Fig. 3g). Circ-0046263 silencing did not affect the expression of miR-133a-5p, and the transfection of miR-133a-5p mimic did not affect the expression of circ-0046263 (Fig. 3h, i). This finding suggests that circ-0046263 acted as miRNA sponges without directly affecting the expression of miRNAs.

### Silencing miR-133a-5p reversed the si-circ-0046263-induced antitumor effects in NPC cells

**Fig. 4:**
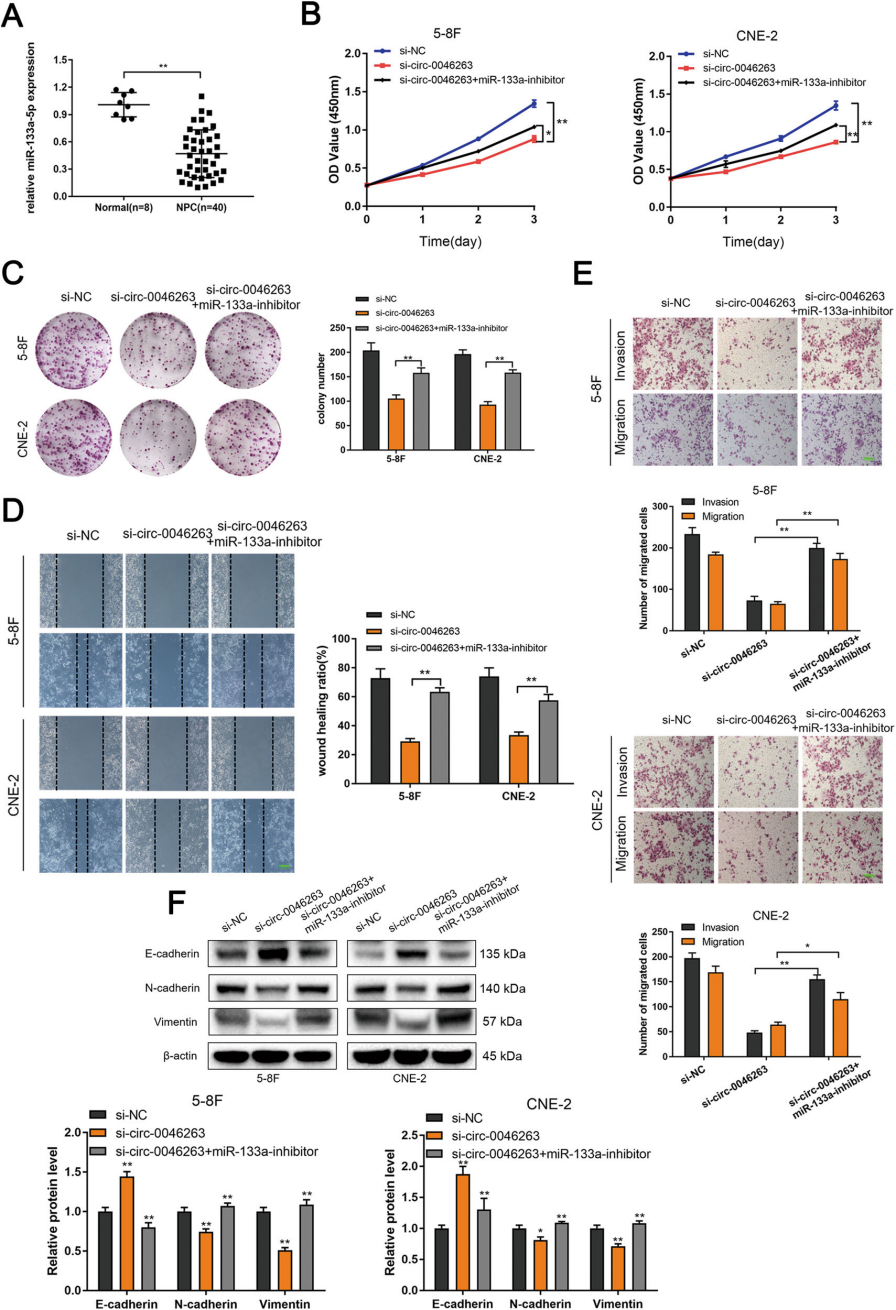
Knockdown of miR-133a-5p reversed the proliferation, migration, and invasion of NPC cells induced by circ-0046263 silencing. **a** qRT-PCR was performed to detect the expression of miR-133a-5p in normal nasopharyngeal epithelial (*n* = 8) and NPC tissues (*n* = 40). **b**, **c** CCK-8 assay and colony formation were performed to assess the proliferative capacity and colony-forming ability of NPC cells transfected with si-NC and si-circ-0046263 with or without the miR-133a-5p-inhibitor. **d** Wound-healing assay evaluated the reversion of migration ability by miR-133a-5p-inhibitor. **e** Migration and invasion abilities were evaluated by transwell assay after indicated treatment. Scale bars, 100 μm. **f** Western blot analysis was performed to detect the relative expression of EMT-related proteins. The data are presented as mean ± SD of at least three independent experiments*.* *P < 0.05; **P < 0.01.

qRT-PCR was performed to examine the miR-133a-5p expression in NPC tissues (Fig. 4a). The results indicated that miR-133a-5p expression was downregulated in NPC tissue samples. miR-133 is a proven “tumor suppressor gene” that is significantly downregulated in solid tumors, such as gastric cancer^14^, lung cancer^15^, and pancreatic carcinoma^16^. Next, we explored the function of miR-133a-5p in NPC cells. As expected, after transfection with miR-133a-5p-inhibitor, the proliferation, migration, and invasion capabilities of cells were increased (Fig. S2A–E). The expression level of E-cadherin was downregulated, and the expression level of N-cadherin and vimentin was upregulated (Fig. S2F), To investigate whether circ-0046263 enhanced the proliferation, migration, and invasion of NPC cells by interacting with miR-133a-5p. We performed rescue experiments and co-transfected with si-circ-0046263 and miR-133a-5p-inhibitor in 5–8F and CNE-2 cells. As shown in Fig. 4b, c, miR-133a-5p inhibition partially attenuated the si-circ-0046263-induced reduction in NPC cell viability as shown by CCK-8 and colony-formation assays. Wound-healing (Fig. 4d) and Transwell assays (Fig. 4e) results indicated that miR-133a-5p downregulation could restore cell invasion and migration. Furthermore, miR-133a-5p inhibition could partially reverse the EMT process impaired by si-circ-0046263 as indicated by the changes in EMT-related protein levels (Fig. 4f). These findings indicated that circ-0046263 promoted NPC progression by eliminating the antitumor effect of miR-133a-5p.

### IGFBP3 is a direct target of miR-133a-5p in NPC

**Fig. 5:**
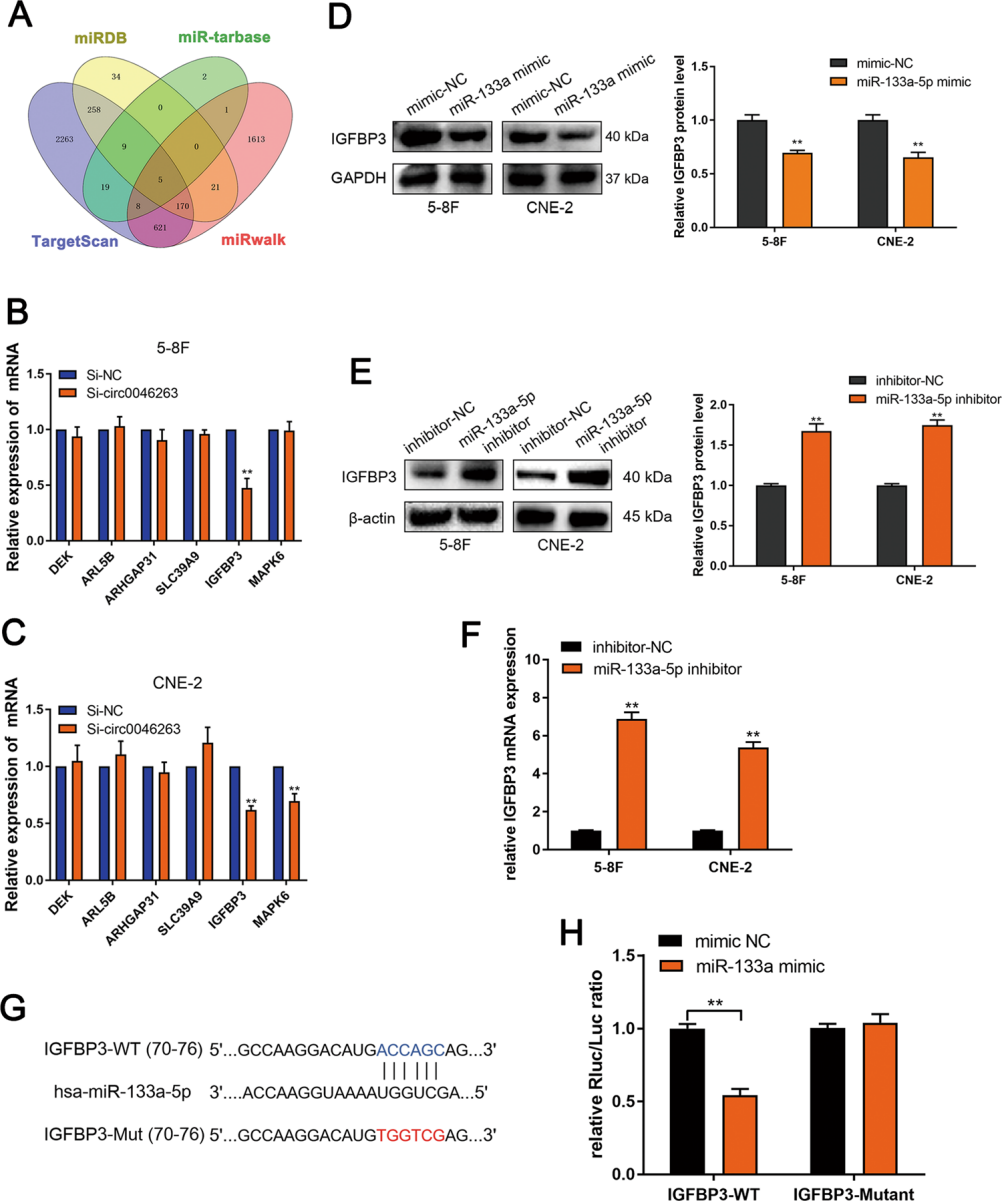
IGFBP3 is a direct target of miR-133a-5p. **a** Schematic diagram showed the overlap of miR-133a-5p target predicted by miRDB, miR-tarbase, TargetScan, and miRwalk. **b**, **c** qRT-PCR was performed to determine the relative expression of six selected genes in 5–8F and CNE-2 cells transfected with si-NC or si-circ-0046263. **d** The protein levels of IGFBP3 in NPC cells transfected with miR-133a-5p mimic or control were evaluated by Western blot analysis. **e** The protein level of IGFBP3 after knocking down miR-133a-5p was detected by Western blot analysis. **f** mRNA expressions of the IGFBP3 was detected by qRT-PCR. **g** Schematic diagram of the complementary sequence between miR-133a-5p and IGFBP3. **h** Dual luciferase reporter assays indicated that miR-133a-5p bound to the 3ʹ-UTR of IGFBP3 and inhibited luciferase activity. Values represent the mean ± SD of three independent experiments. *P < 0.05; **P < 0.01.

To elucidate which target gene was regulated by miR-133a-5p in NPC. Targetscan, miRDB, miR-tarbase, and miRwalk were used to predict target genes (Fig. 5a). MAPK6 is a known target gene of miR-133a-5p^17^. We comprehensively identified six target genes (DEK, ARL5B, ARHGAP31, SLC39A9, IGFBP3, and MAPK6), and their mRNA levels were detected after silencing circ-0046263 in 5–8F and cne-2 cells. Only IGFBP3 was downregulated in both cells (Fig. 5b, c). Then, we observed a substantial decrease in the protein level of IGFBP3 after the transfection of miR-133a-5p mimics in 5–8F and cne-2 cells (Fig. 5d). In addition, we transfected the inhibitor of miR-133a-5p in 5–8F and CNE-2 cell lines, and detected that the protein and mRNA levels of IGFBP3 increased after transfection (Fig. 5e, f). Furthermore, we cloned the Wt and Mut 3ʹ-UTR of IGFBP3 mRNA and performed a dual luciferase reporter assay (Fig. 5g). The miR-133a-5p mimics effectively inhibited luciferase activity of the wild type compared with that of the control group. However, luciferase activity was restored after the binding sites were mutated (Fig. 5h). Thus, we confirmed that IGFBP3 is the direct target of miR-133a-5p.

### Circ-0046263 promoted NPC progression via the circ-0046263-miR-133a-5p-IGFBP3 axis

**Fig. 6:**
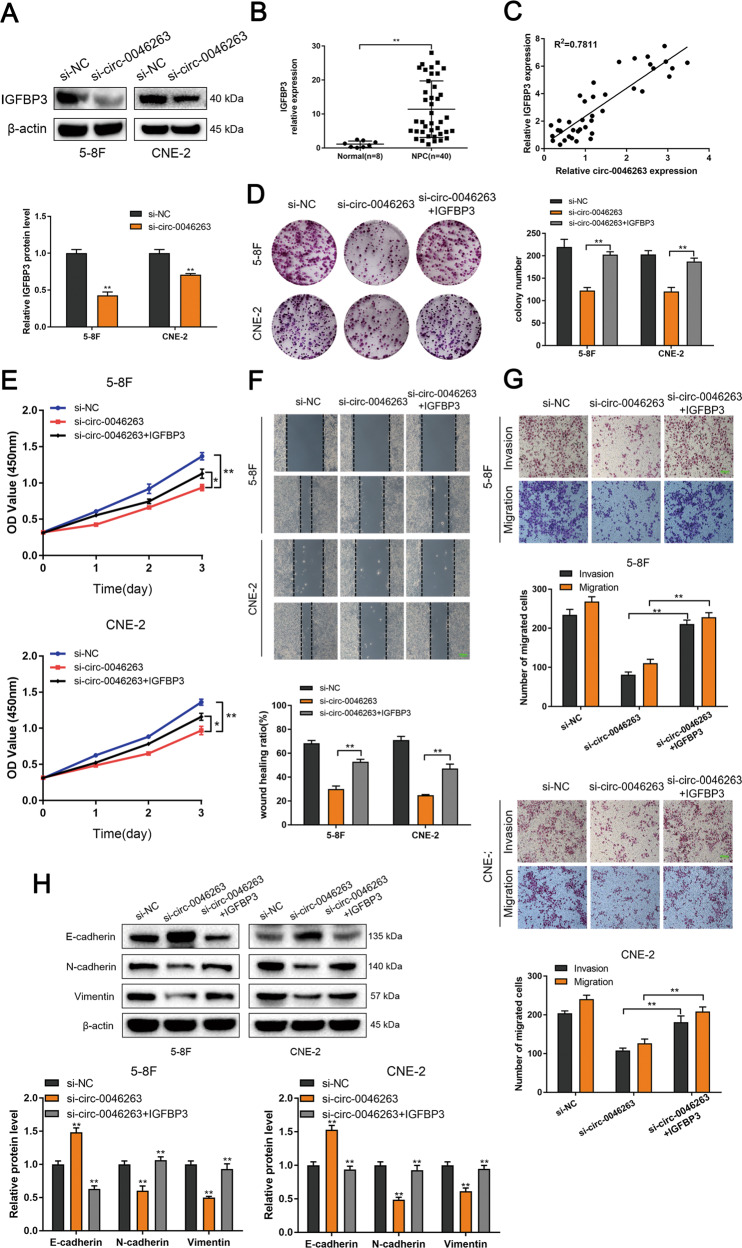
circ-0046263 promoted NPC cell progression by modulating IGFBP3 expression. **a** The expression level of IGFBP3 after knocking down circ-0046263 was detected by Western blot analysis. **b** qRT-PCR assay was performed to detect the expression of IGFBP3 in normal nasopharyngeal epithelial (*n* = 8) and NPC tissues (*n* = 40). **c** Correlation analysis revealed that IGFBP3 expression was positively correlated with circ-0046263 expression in NPC tissues. **d**, **e** IGFBP3 overexpression restored circ-0046263 knockdown-mediated cell viability and growth by CCK-8 assay and colony-formation assay. **f**, **g** Overexpression of IGFBP3 rescued the effect of circ-0046263 knockdown on cell invasion and migration. The effect was evaluated by wound-healing and transwell assays. Scale bars, 100 μm. **h** Western blot analysis of EMT-related protein expression. Values represent the mean ± SD of three independent experiments. *P < 0.05; **P < 0.01.

To verify whether circ-0046263 promotes NPC progression via the circ-0046263-miR-133a-5p-IGFBP3 axis, we first demonstrated a decreased in the protein level of IGFBP3 after silencing circ-0046263 (Fig. 6a). The mRNA level of IGFBP3 was upregulated in 40 NPC patients and positively correlated with the expression level of circ-0046263 (Fig. 6b, c). We constructed an IGFBP3 overexpression plasmid and transfected it into circ-0046263-silenced 5–8F and CNE-2 cells. Compared with the cells transfected with si-circ-0046263, the colony-forming ability of the cells cotransfected with the IGFBP3 overexpression plasmid was restored (Fig. 6d). In addition, IGFBP3 overexpression restored the proliferation ability of NPC cells as shown by the CCK-8 analysis (Fig. 6e). As expected, the migration and invasive abilities were significantly rescued by IGFBP3 (Fig. 6f, g). The changes in EMT-related protein levels also indicated that IGFBP3 reverses EMT impairment caused by circ-0046263 knockdown (Fig. 6h). Taken together, these findings indicated that circ-0046263 primarily promoted the progression of NPC cells by targeting IGFBP3.

### Tumor growth and metastasis regulation by circ-0046263 in NPC in vivo

**Fig. 7:**
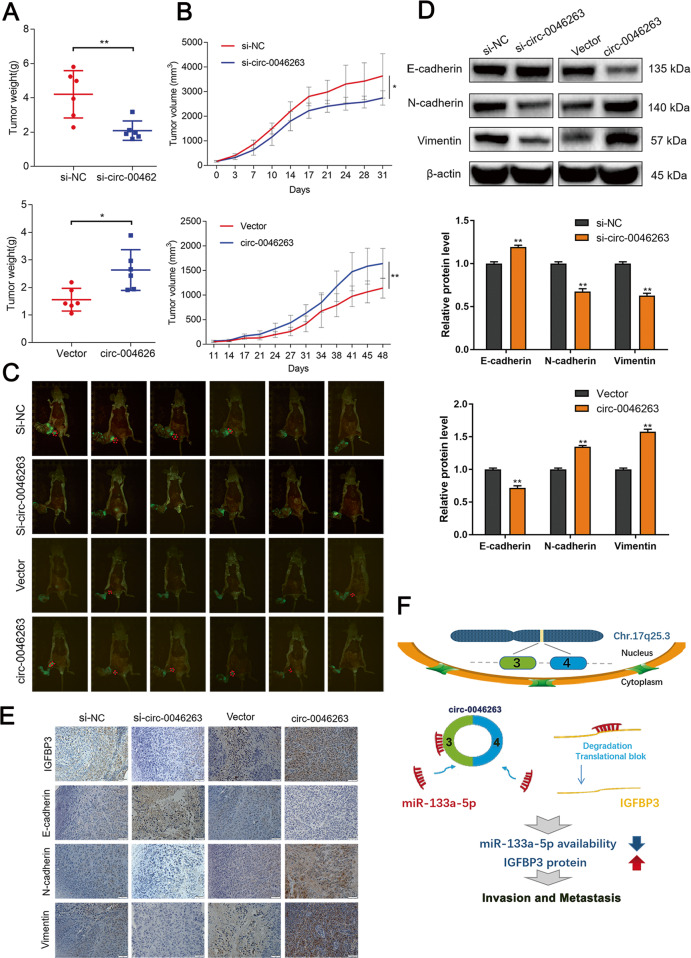
Circ-0046263 regulated tumor growth and metastasis in vivo. **a,** **b** The mean tumor weight (g) and size (mm^3^) were analyzed. **c** Images of xenograft tumors in four different nude mouse groups. The red circle indicates lymph node metastasis in nude mice. **d** Expression of EMT-related proteins was detected by Western blot analysis. **e** Expression of IGFBP3, E-cadherin, N-cadherin, and vimentin were determined by immunohistochemistry. **f** Schematic illustration of the circ-0046263/miR-133a-5p/IGFBP3 axis. Values represent the mean ± SD of three independent experiments. *P < 0.05; **P < 0.01.

Basing on the in vitro results, we further validated the effect of circ-0046263 on NPC in vivo. We established a xenograft tumor model in nude mice using circ-0046263 suppressing or overexpressing NPC cells. After building the model, we regularly measured the quality of the mice, and we found no significant difference between the experimental group and the control group (Fig. S3). The results indicated that circ-0046263 knockdown significantly delayed the tumor growth of the mice, whereas those treated with highly expressed circ-0046263 had the largest tumor (Fig. 7a, b). After 6 weeks, the mice were sacrificed, and popliteal lymph node metastasis was observed after necropsy. Compared with the respective controls, the number of mice with lymph node metastasis decreased in the si-circ-0046263 group but increased in the circ-0046263 overexpression group (Fig. 7c and Table S2). We extracted proteins from the tumors, and Western blotting shown changes in EMT-related protein expression levels (Fig. 7d). In addition, IHC was further used to assess the expression of E-cadherin, N-cadherin, vimentin, and IGFBP3 in tumors. Compared with the corresponding control group, E-cadherin was significantly increased in the si-circ-0046263 group, whereas IGFBP3, N-cadherin, and vimentin were increased in the circ-0046263 overexpression group (Fig. 7d, e). These results confirmed that circ-0046263 promoted NPC proliferation, migration, and invasion in vivo.

## Discussion

Comprehensive treatment of radiation therapy and chemotherapy has greatly improved the outcome of NPC. The 5-year overall survival rate of patients with NPC has increased in the past decades^18^. However, distant metastasis is still the major cause of mortality in NPC. Distant metastasis and tumor recurrence remain refractory to treatment. Novel targets and treatments that are effective for advanced stages of NPC are urgently needed.

Increasing evidence indicates that circRNAs are dysregulated in multiple tumors and may be involved in EMT, metastasis, and tumorigenesis^6,19^. A previous study revealed that circHIPK3 acts as miRNA sponge of miR-4288 to upregulate ELF3 and promotes proliferation and invasion of NPC cells^20^. Zhong et al. found that circular RNA CDR1as can sponge miR-7-5p, thereby enhancing E2F3 stability and promoting the growth of NPC^21^. However, the function of deregulated circRNAs in NPC still remains largely unknown.

Herein, we showed that circ-0046263 was significantly upregulated in NPC tissues and several NPC cell lines, indicating that circ-0046263 is a potential cancer-related gene in NPC. CircRNA circ-0046263 knockdown suppressed NPC cell growth, migration, EMT, and metastasis in vitro and in vivo, indicating that circRNA circ-0046263 has an oncogenic role in NPC.

Emerging evidence indicates that circular RNA may be an attractive treatment target for cancer metastasis. Previous studies suggest that circRNAs may function as miRNA sponges, translate to proteins, or interact with RNA-binding protein to regulate their downstream targets^22,23^. Our FISH and nuclear/cytoplasmic fraction analyses revealed that circ-0046263 was mainly located in the cytoplasm, indicating that it may act as miRNA sponges. We conducted bioinformatics analysis, which revealed that circ-0046263 had multiple potential binding sites for a number of miRNAs. Our RIP and dual luciferase reporter assays revealed the direct binding of miR-133a-5p to circ-0046263. Circ-0046263 exerted its function by a ceRNA mechanism, competitively bound to miR-133a-5p, and then abolished the suppressive effect of miR-133a-5p on its target gene IGFBP3.

IGFBP3, or insulin-like growth factor (IGF)-binding protein 3, also known as IBP3, is a member of the IGFBP family. IGFBP3 can prolong the half-life time of IGFs and is involved in IGF pathways. IGFBP3 also exhibits IGF-independent effects. Previous studies have shown that IGFBP3 expression is correlated with various cancers, such as esophageal cancer^24,25^, gastric cancer^26^, colorectal cancer^27,28^, pancreatic cancer^29^, prostate cancer^30–32^, and lung cancer^33,34^. Previous studies have reported that IGFBP3 expression is increased in many tumors with metastasis, including lung cancer and oral cancer^35,36^. According to Bao et al.^37^, upregulation of IGFBP3 promoted cell proliferation, invasion, and metastasis in NPC, and IGFBP3 was significantly elevated in NPC and its expression level was correlated with TNM stage of patients. These results are consistent with our findings.

EMT is a crucial step in tumor cell invasion and metastasis. EMT process is marked by the suppression of epithelial markers E-cadherin and expression of mesenchymal markers N-cadherin^38^. IGFBP3 has been shown to facilitate EMT process and play an important role in the TGF-β pathway. Yang et al.^39^ demonstrated that upregulation of IGFBP3 promoted lung cancer cell EMT process, migration, and invasion. Natsuizaka^40^ reported that IGFBP3 enhanced TGF-β-mediated EMT process and upregulated transcription factors essential for EMT by allowing persistent activation of SMAD2/3. In the current study, we observed the effect on the activation of SMAD2 and SMAD3 by knocking down or overexpressing circ-0046263. The results show that circ-0046263 could promote the activation of SMAD2 and SMAD3 (Fig. S4A, B). In addition, we observed that knockdown of circRNA circ-0046263 increased the expression of E-cadherin and suppressed the expression of N-cadherin in the NPC cells, whereas IGFBP3 overexpression could reverse this process. Our results suggested that the circ-0046263/miR-133a-5p/IGFBP3 axis facilitated NPC metastasis via EMT.

Although the upregulation of IGFBP3 by circRNA circ-0046263 may mediate NPC proliferation and metastasis, more details about IGFBP3 in EMT process are still needed. In addition, more NPC cell lines will be used in our next stage of studies to verify the detailed metastatic effects and mechanism of IGFBP3 in NPC.

The data we presented here showed new light on the possible mechanisms of circRNA regulating NPC proliferation and metastasis. In summary, we found that circRNA hsa-circ-0046263, which was upregulated in NPC tissues and NPC cell lines, could sponge miR-133a-5p, upregulated the expression of downstream targets gene IGFBP3, and promoted NPC cell migration, invasion, and tumor metastasis (Fig. 7f). In addition, the circ-0046263/miR‑133a-5p/IGFBP3 axis promoted metastasis involving an EMT process. Our study enriches the research on the molecular mechanism of circRNA involved in the invasion and metastasis of NPC and provides new ideas for therapeutic targets in clinical cancer therapy.

## Supplementary information

Supplementary Tables and Figure

Supplementary Figure

Supplementary Figure

Supplementary Figure

Supplementary Figure

Supplementary Tables S1

Supplementary Tables S2
