# Supplementary material for: Hsa_circ_0046263 functions as a ceRNA to promote nasopharyngeal carcinoma progression by upregulating IGFBP3
Source: Cell Death Dis. 2020 Jul 23;11(7):562. doi: 10.1038/s41419-020-02785-3 (PMC7378203; doi:10.1038/s41419-020-02785-3)
Supplement: Supplementary file 1 — Supplementary Tables and Figure [file 41419_2020_2785_MOESM1_ESM.docx]

**Supplementary Tables and Figure:**

Supplementary Table S1: Primers and siRNAs sequences used in this work.

Supplementary Table S2: Lymph node metastasis rate of xenograft tumors (n=6 per group)

Supplementary Figure S1: Overexpression of circ-0046263 promoted proliferation, invasion, and migration of NPC cells in vitro.

Supplementary Figure S2: MiR-133a-5p regulated the migration, invasion and proliferation of NPC cells.

Supplementary Figure S3: The quality of nude mice in experimental group and control group.

Supplementary Figure S4: Circ-0046263 promoted SMAD2 and SMAD3 activation.

**Figure S1** Overexpression of circ-0046263 promoted proliferation, invasion, and migration of NPC cells in vitro. (A) The circ-0046263 expression in stable SUNE-1 cell clones infected with lentiviruses encoding circ-0046263. (B) Colony-formation assay was used to evaluate the colony-forming ability of SUNE-1 cells. (C) CCK8 assay was performed to determine the proliferation capacities of SUNE-1 cells. (D and E) Wound-healing and Transwell assays reflected migratory and invasive capacities of NPC cells. (F) Expression of EMT-related proteins was detected by Western blot analysis. (G) circ-0046263 induced morphological changes in NPC cells. Scale bars, 100 μm. The data were from one representative experiment among three independent experiments and are expressed as mean +SD. *P* <0.05 (*), *P*<0.01(**).

**Figure S2** MiR-133a-5p regulated the migration, invasion and proliferation of NPC cells. (A) qRT-PCR was performed to detect the expression of miR-133a-5p. (B) CCK8 assay was performed to determine the proliferation capacities of 5-8F and CNE-2 cells. (C) The proliferative capacity in 5-8F and CNE-2 cells were determined via colony formation assay. (D) Wound-healing assay evaluated migration ability. (E) Migration and invasion abilities were evaluated by Transwell assay. Scale bars, 100 μm. (F) Western blot analysis was performed to detect the relative expression of EMT-related proteins. The data are presented as mean+SD of at least three independent experiments. *P* <0.05 (*), *P*<0.01(**).

**Figure S3** The quality of xenograft tumor model nude mice. (A) Quality records of si-circ0046263 group and control group. (B) Quality records of circ0046263 overexpression group and control group.

**Figure S4** Circ-0046263 promoted SMAD2 and SMAD3 activation. (A) Western blot analysis was performed to detect the relative expression of Smad2 and Smad3 after transfection with si-circ-0046263. (B) Western blot analysis of Smad2 and Smad3 expression after overexpression of circ-0046263. Values represent the mean+SD of three independent experiments. *P* <0.05 (*), *P*<0.01(**).
