# Supplementary figures and images for: Hsa_circ_0046263 functions as a ceRNA to promote nasopharyngeal carcinoma progression by upregulating IGFBP3
Source: Cell Death Dis. 2020 Jul 23;11(7):562. doi: 10.1038/s41419-020-02785-3 (PMC7378203; doi:10.1038/s41419-020-02785-3)

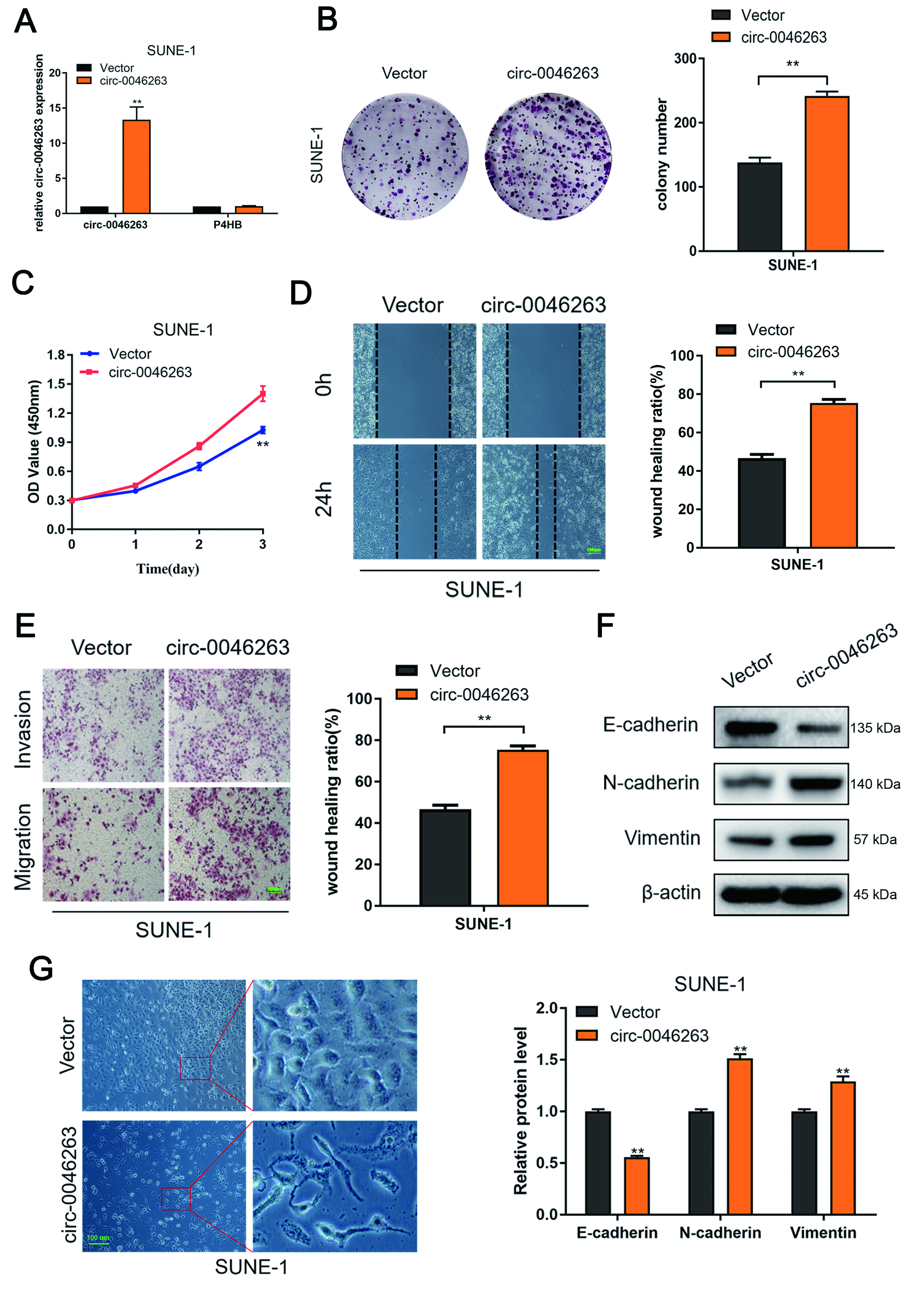

Supplement: Supplementary file 2 — Supplementary Figure [file 41419_2020_2785_MOESM2_ESM.tif]

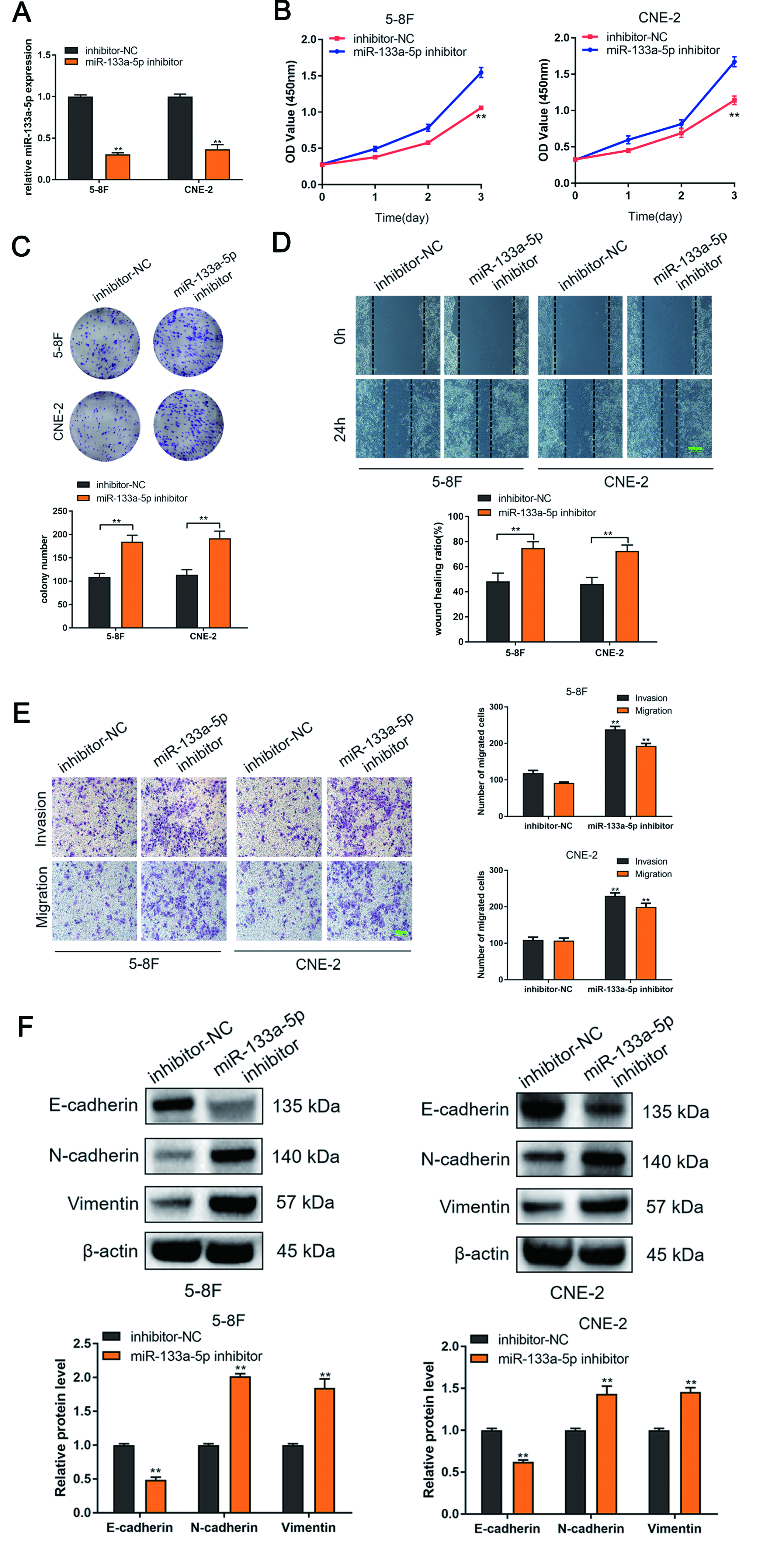

Supplement: Supplementary file 3 — Supplementary Figure [file 41419_2020_2785_MOESM3_ESM.tif]

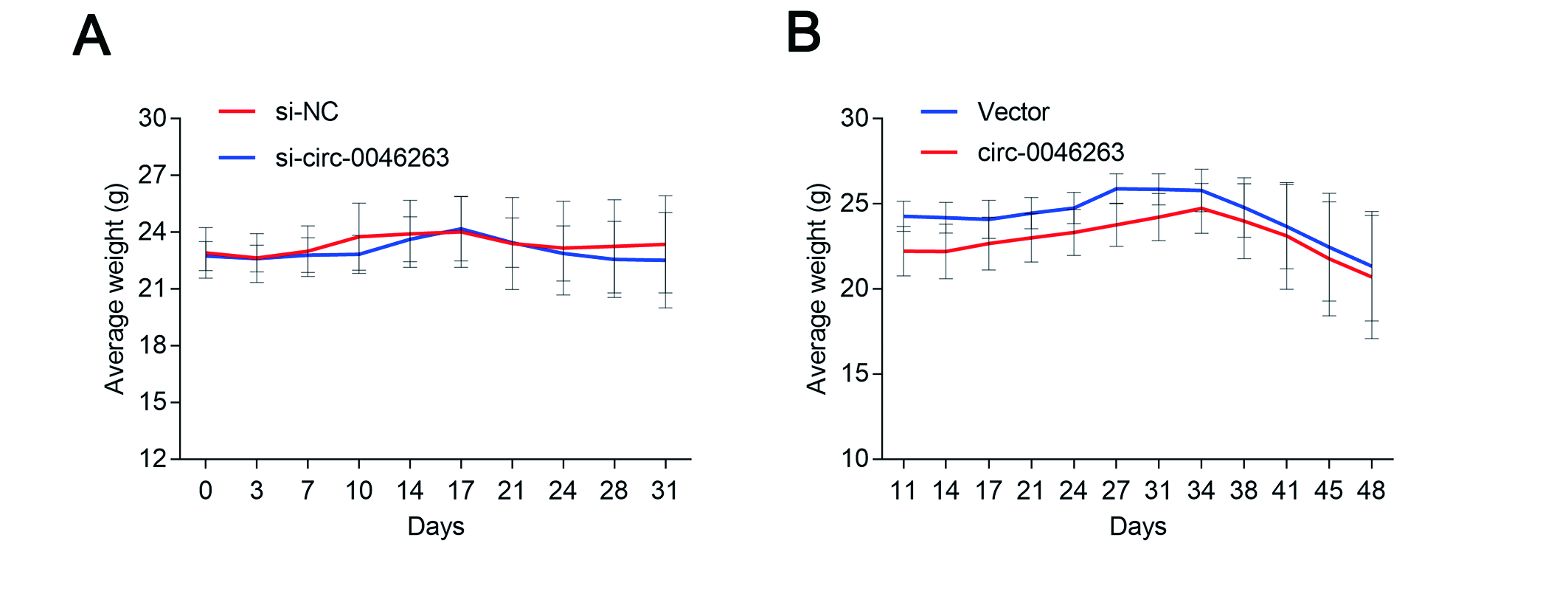

Supplement: Supplementary file 4 — Supplementary Figure [file 41419_2020_2785_MOESM4_ESM.tif]

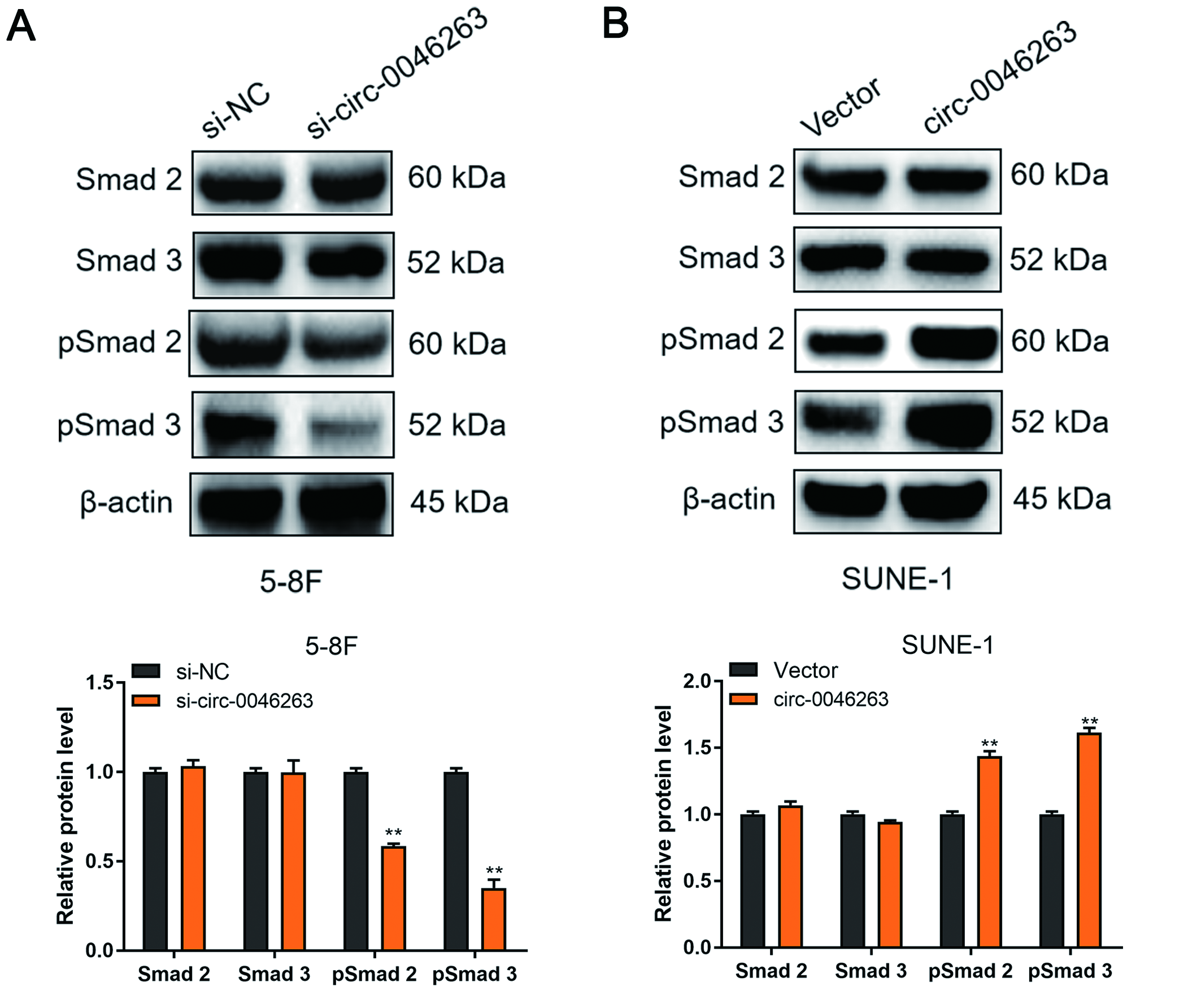

Supplement: Supplementary file 5 — Supplementary Figure [file 41419_2020_2785_MOESM5_ESM.tif]
